# The Development of Innovative Dosage Forms of the Fixed-Dose Combination of Active Pharmaceutical Ingredients

**DOI:** 10.3390/pharmaceutics14040834

**Published:** 2022-04-11

**Authors:** Magdalena Janczura, Szymon Sip, Judyta Cielecka-Piontek

**Affiliations:** 1 Synteza sp. z o.o., św. Michała 67/71, 61-005 Poznań, Poland; magdalena.janczura@synteza.com.pl; 2 Department of Pharmacognosy, Faculty of Pharmacy, Poznań University of Medical Sciences, Rokietnicka 3, 60-806 Poznań, Poland; szymonsip@ump.edu.pl

**Keywords:** fixed-dose combination (FDC), bilayer, multilayer tablets, gastric retention of oral dosage forms, hot-melt extrusion, three-dimensional (3D) printing, history of innovative forms

## Abstract

The development of innovative forms of combination drugs is closely related to the invention of the multilayer tablet press, polymers for pharmaceutical applications, the hot-melt extrusion process, and 3D printing in the pharmaceutical industry. However, combining multiple drugs within the same dosage form can bring many physicochemical and pharmacodynamic interactions. More and more new forms of fixed-dose combinations (FDCs) have been developed due to work to overcome the incompatibility of active substances or to obtain different drug release profiles in the same dosage form. This review provides discussions of the application of various innovation formulation technologies of FDC drugs such as bilayer system, multilayer tablet, active film coating, hot-melt extrusion, and 3D printing, taking into account the characteristics of the key ingredients in the FDC formulation and presenting technological problems and challenges related to the development of combination drugs. Moreover, the article summarizes the range of dosage forms that have been made using these technologies over the past 30 years.

## 1. Introduction

For the last few decades, the pharmaceutical industry has been researching the potential of improving the currently available medicinal products, including increasing the safety and effectiveness of their use or reducing the side effects of therapy. The work carried out in this direction also aims to increase patients’ access to modern therapies and facilitate patients’ compliance with their doctor’s instructions. In particular, new drug applications (forms, administration routes) and more cost-effective and improved technologies for their production are being developed. The importance of complex medicinal products combining two or more active substances is growing. Such products aim to increase the patient’s tolerance of the drug, reduce side effects, facilitate patient–doctor cooperation, and sometimes increase the drug’s effectiveness. Some studies show that taking combined products (combinations of active substances) may be more effective than using individual active substances in medicinal products. Although these improvements may seem like a simple modification of existing drugs, developing such changes while preserving the drugs’ properties takes time and considerable expense. Patients benefit from this as they have safer, easier to apply, and more “friendly” pharmaceuticals at their disposal.

An example may be formulations for new combinations (two or more active substances in one tablet), introduced by the pharmaceutical industry, such as:

- Hard capsules containing pellets, mini-tablets, microcapsules, or the encapsulation of liquid combinations;
- Bilayer tablets;
- Multilayer tablets;
- Gastric retention of oral dosage forms (floating capsules, hydrodynamically balanced systems, raft-forming systems, expandable);
- Delivery systems by hot-melt extrusion, co-extrusion;
- Three-dimensional (3D) printing technology.

This article gives an overview for pharmaceutical technologists on the latest trends in FDC formulations and shows how to select the most optimal formulation strategy using examples from the history of innovative combination drug formulations.

## 2. Advantages and Disadvantages of Using FDCs

Despite the growing popularity of FDC-type drugs and a significant increase in the formulations available on the market for various disease entities, FDC-type drugs have drawbacks that limit their use or raise some controversies related to the risk for the patient [1,2]. However, as with any innovative solution, special attention should be paid to whether the advantages outweigh the disadvantages, which we see in the case of FDCs. The observed benefit in favor of their use is confirmed by the increasing number of formulations on the market and the frequency of their use in patients, particularly in chronic diseases requiring multi-drug therapy (Table 1).

## 3. Hard Capsules in Fixed-Dose Combinations

Developed in the 19th century, the two-piece hard capsule is still a popular dosage form in the pharmaceutical industry; moreover, it is a suitable dosage form for delivering fixed-dose combinations drugs (FDCs). The first patent for producing capsules and their capsules was issued in France in 1834. These were hard gelatin capsules. Their production technology was to solidify hot liquid gelatin on a cold brass rod. The capsules quickly gained popularity in France and abroad. In 1835, France alone used 3.5 tons of gelatin [3]. There are two main types of capsules used as dosage forms in the pharmaceutical industry: gelatin and hydroxypropyl methylcellulose (HPMC) capsules [4]. Gelatin capsules are conventionally produced using animal sources. They deliver fast dissolution in all biological media, wherein the drug release is achieved in 5–10 min. HPMC is a plant-origin capsule with better stability and flexibility than gelatin capsules. The advent HPMC has facilitated the filling of hygroscopic ingredients, a challenge with traditional gelatin capsules. The combined capsule products have evolved naturally due to advances in capsule filling technology. In addition to powder filling, today, formulation scientists can achieve a combination of liquid capsule filling. It is possible to mix the spheres and pellets with liquid or to put small capsules filled with liquid into larger capsules filled with liquid. Many successful FDC products use hard capsules as drug containers. Here are some examples:

### 3.1. Encapsulation of Liquid Combination

**Figure 1:**
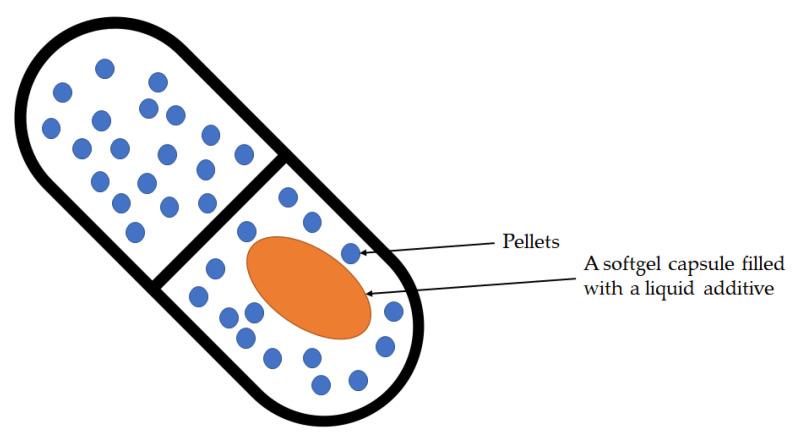
The structure of the Combodart/Jalyn: A softgel capsule filled with a liquid additive. The above softgel capsule was introduced into a hard size-00 HPMC capsule. Pellets from the other additive fill the HPMC capsule to accompany the softgel in the final dosage form.

Combodart/Jalyn was the first FDC designed to treat moderate to severe benign prostatic hyperplasia (BPH) and reduce the risk of acute urinary retention in BPH-related surgery [5]. The product consists of dutasteride, an alpha-reductase inhibitor, and an alpha-blocker, tamsulosin hydrochloride. Dutasteride delays the progression of BPH by inhibiting the production of dihydrotestosterone, the hormone that stimulates the growth of the male prostate, while tamsulosin provides quick symptom relief by reducing smooth muscle tone in the prostate and bladder neck. The structure of the product as part of the delivery system is shown in Figure 1.

### 3.2. Cardiovascular Polypill

A polypill is a drug product in a pill (i.e., capsule or tablet) that combines multiple active pharmaceutical ingredients. The prefix “poly” means “many”, referring to many different drugs in a given “pill”. An occasional synonym is combopil. It is commonly manufactured as a fixed-dose medicinal product (FDC) to treat or prevent chronic disease. The term “polypill” was first used to prevent cardiovascular disease [6,7,8] but has since gained wider acceptance, including combinatorial drugs that existed before the term was used.

The first combination drugs for hypertension that appeared in the 1950s included hydralazine or reserpine. In the 1960s, drugs containing a combination of methyldopa and a diuretic, and reserpine and a diuretic, were introduced [9]. They demonstrated high antihypertensive efficacy and, at that time, constituted a valuable supplement to the treatment of arterial hypertension. However, they were withdrawn due to frequent and severe side effects. We had to wait several years for other combination drugs to follow.

Over the past decade, tremendous efforts have been made to develop a variety of cardiovascular polypills in response to the global rise in cardiovascular disease. In 2001, experts from the World Health Organization and the Wellcome Trust discussed interventions in noncommunicable diseases and noted that taking one pill can encourage patient compliance and seriously reduce drug costs [10]. In 2002, the annual report of the World Health Organization identified a significant potential impact on public health and cost-effectiveness of increasing access to combined cardiovascular treatment [11]. The choice of drugs to be included in the combined pill is a multi-step process. A Lancet article outlined that a four-component combination pill would reduce cardiovascular risk by about 75% among people with vascular disease [12]. Wald and Law, in 2003, suggested a pill consisting of six different compounds to maximize the potential benefits. They presented a statistical model that suggested how widespread use of such a polypill could reduce mortality from heart disease and stroke by up to 80%, while using drugs whose interactions are already relatively well known and understood due to many years of prescribing them together [6,7].

**Figure 2:**
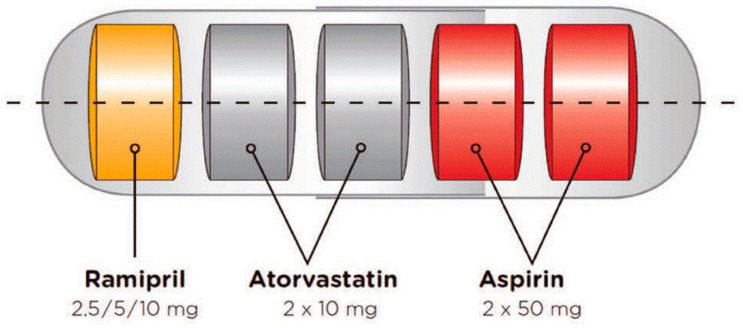
The Fuster-CNIC-Ferrer CV Polypill (Trinomia ^®^, Sincronium ^®^, Iltria ^®^) technology [13,14].

The Fuster-CNIC-Ferrer CV Polypill as a multi-layered formulation, housed in a capsule, was developed using a technology patented by Ferrer [13]. Ferrer scientists began work on the polypill in 2007, and the first pill was launched in 2014. The cardiovascular polypill consists of aspirin (100 mg), ramipril (2.5, 5, or 10 mg), and atorvastatin (20 mg) in a capsule. Studies have demonstrated that treating with polypills in patients with established cardiovascular disease reduces mortality [4,12]. The technology successfully avoids the physicochemical incompatibilities of the individual components and conserves their biopharmaceutical and pharmacokinetic properties (Figure 2).

Another example is the CV pill (Torrent Pharmaceuticals) available in a capsule and tablet kit. The capsule contains a statin (Atorvastatin 10 mg), an ACE inhibitor (Ramipril 5 mg), and an anti-platelet agent (Enteric-coated Aspirin 75 mg). The tablet contains a beta-blocker (Metoprolol Succinate extended-release 50 mg).

Despite introducing the polypill concept consisting of a combination of cardiovascular products sold almost 15 years ago, progress in this area was slow, and only one product was approved in a relatively wide geographical area (Trinomia Ferrera). Some cardiovascular polypills are already available in some countries in Europe, South America, and India, and the FDA and EMA are reviewing applications (see examples in Table 2).

Polypills are a valuable therapeutic tool for people suffering from various diseases due to the combination of many drugs in one product, thus, simplifying the administration of drugs to medical personnel and relieving the patient. Moreover, elderly patients may require several medications daily to manage many conditions and are particularly prone to difficulties remembering or following their regimen. Occasionally, multiple drugs in a given pill may target one underlying condition or a group of related conditions as this widens the pool of potential patients for whom a given drug combination may be appropriate.

### 3.3. Inhalation Combinations

Capsules are considered the most suitable dosage form for FDCs [18]. They are inert and available in multiple sizes. Hard capsules can be used to inhale drugs, where the capsule is punctured, and the patient inhales to help the powder reach the lungs [19]. In capsule-based dry powder inhalation technology, combination therapy produces better results than a single ingredient. A good example is a formoterol-budesonide mixture [20].

### 3.4. Fixed-Dose Combination Softgel

**Figure 3:**
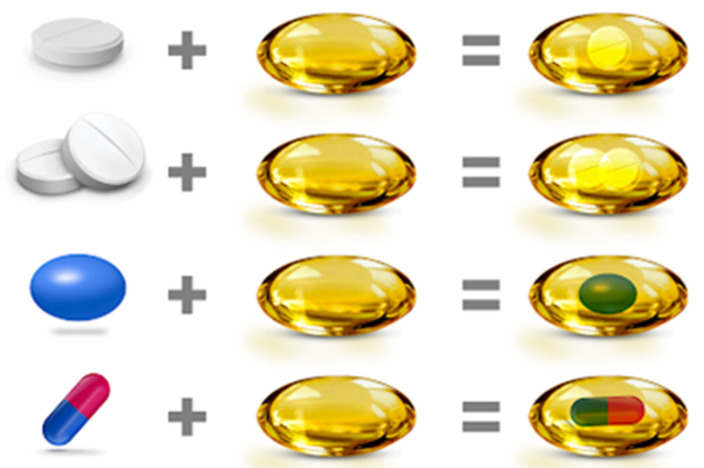
Soft gelatin capsules for fixed-dose combination drugs—Unigel™ technology that allows the use of other delivery systems, such as tablets, capsules, micro granules, or pellets, closed in one soft capsule, the so-called “softgel” [21].

Unigel™ is a patented fixed-dose combination oral dosage form where tablets, granules, pellets, or capsules are filled into a large, soft gelatin capsule (Figure 3). The advantage of this technology is the ability to combine active ingredients with different release profiles or with chemical incompatibility problems. In addition, ingredients may be combined, at least one in a liquid or semi-solid state.

## 4. Bilayer Tablets

**Figure 4:**
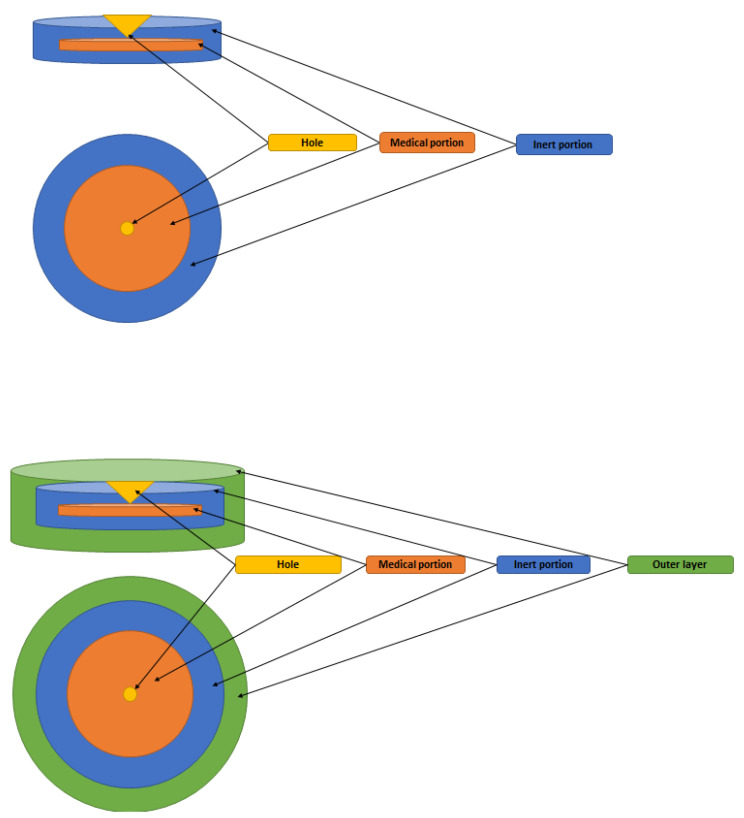
Bilayer tablet according to the Stephenson patent from 1964 [23].

In 1843, the 19th-century British writer, painter, and inventor William Brockedon patented a device for compressing sodium carbonate and potassium carbonate in the form of tablets and lozenges (Figure 4). The introduction of compressed tablets to medicine by him was one of the most far-reaching achievements in the history of drug production [22]. Today, tablets are the most common and widely used form of drug dosage. Further development of powder compression technology resulted in tablets consisting of several layers.

The history of bilayer tablets is relatively older, over 50 years—one of the early scientific evaluations of layered tablets was published by Stephenson [23].

Gunsel et al., designed a method in the 1970s to check the weight of individual layers by taking samples without stopping the machine, ensuring the machine is checked for correct dosing. Considerable expertise was required to modify this method further to produce double-layer tablets and to ensure consistent production to meet the regulatory requirements. It was concluded that the formulations used for every layer should themselves be compressible and compactable, i.e., they should show a satisfactory reduction in volume and form mechanically strong, coherent solids. On this assumption, the interface between the layers should weld during compaction, and the strong adhesion forces should hold the layers together after tablet ejection [18,19]. The observations were first confirmed by Karehill et al., who described that the compaction pressure used to form the first tablet layer should be kept to a minimum to provide sufficient surface roughness for the particles to nest and engage between the layers. Due to the increase in surface roughness, there is a larger contact area between the layers, which increases the interlayer. Attention was drawn to the need to develop an experimental method that could be applied to bilayer tablets to detect lamination tendencies that are no longer visible when the tablet is ejected but which only manifest after storage and handling of the compacts [24].

A fixed-dose two-layer tablet with high dose metformin hydrochloride in an extended-release layer and low dose evogliptin tartrate in the immediate-release layer was developed by Dong Han Won et al., based on the Quality by Design (QbD) concept, which assumes that quality should be “built-in” to the product through a well-thought-out and adequately designed, and then constantly monitored, technological process [25].

Kumar Dinakaran et al., developed a combination of ziprasidone hydrochloride NR 40 mg and trihexyphenidyl hydrochloride SR 4 mg in sustained release bilayer floating tablets. The tablets were prepared using sodium HPMC K4M/HPMC K15M as bioadhesive polymers and sodium bicarbonate as a floating layer [26]. Commercial examples of bilayer tablets are shown in Table 3.

Advantages of the bilayer tablet dosage form are [37,38,39,42,43]:

- These are unit dosage forms with the most remarkable capabilities of all oral dosage forms for dose precision.
- Maximum prevention of cross-contamination between the two layers.
- Clear visual separation between the two layers.
- Low compression force to avoid capping and separating the two individual layers.
- Lowered cost compared to all other oral dosage forms.
- Easiest and cheapest to package and strip.
- Lighter and compact.
- Easy to swallow with the slightest tendency for hang-up.
- Product identification is easy and rapid, requiring no additional label when employing an embossed and/or monogrammed punch face.
- Suitable for large-scale production.

## 5. Multilayer Tablet

**Figure 5:**
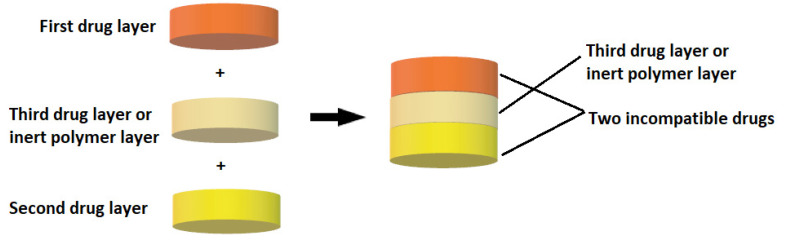
Diagram of a multilayer tablet structure.

Multilayer tablets represent a new era in developing innovative controlled release formulations with different functions for efficient drug delivery (Figure 5). Bilayer tablets can be a crucial option for avoiding chemical incompatibilities between active pharmaceutical ingredients (APIs) by physically separating them and facilitating different drug release profiles. Many pharmaceutical substances are incompatible with each other, but in the case of multilayer tablets, formulators can insert an inert barrier layer between incompatible matrices to prevent interactions. The multilayer tablet is suitable for the chronological release of two drugs from one pharmaceutical form and for obtaining a sustained drug release where, e.g., one layer serves as an immediate release as a loading dose, and the other layer gradually releases the maintenance dose. The use of bilayer tablets is an utterly innovative aspect in the case of antihypertensive, anti-diabetic, anti-inflammatory, or analgesic drugs, in which combination therapy is often used. Pharmaceutical companies are currently developing bilayer tablets for various reasons, such as improving the effectiveness of therapy, patent extension, or marketing purposes (Table 4).

Multilayer tablets are designed for a variety of reasons:

- Separation of incompatible active pharmaceutical ingredients (APIs).
- To control the rate of release of one or two different active pharmaceutical ingredients.
- Controlling the release of an API from one layer by taking advantage of the functional properties of the other layer (such as osmotic properties).
- To modify the total surface area of the API layer utilizing a spacer with one or two inactive layers to obtain swollen or erodible modified-release barriers.
- Possibility to implement fixed-dose combination drugs (FDCs), develop new drug delivery systems, and thus, extend the life cycle of a medicinal product (patent extension).

## 6. Oral Sustained Release Dosage Forms

It has long been a goal of the pharmaceutical industry to develop oral sustained release dosage forms for ease of administration, better adhesion, improved pharmacokinetics, and reduced dosing frequency [53,54]. However, in oral medicaments, all of the commercially available gastric-resident dosage forms, most of them in development, are limited to a gastric residence of less than one day.

In the 1980s, Cargill et al., demonstrated, for the first time, a consistent 24 h gastric retention of oral dosage forms with the release controlled by erosion in a beagle dog [55]. This design used a decomposable tetrahedral dosage form composed of erodible polymers. The tetrahedral dosage form passes from the stomach in about 12 h in humans.

## 7. Floating Capsules

A particular problem arises when a long-acting drug is planned, and the absorption of the drug substance is limited by the site of absorption from the gastrointestinal tract. In order to guarantee the absorption for the required time, it is necessary to use a technology where the drug substance is slowly and evenly released at the site of action. This is where absorption occurs in the stomach or the upper small intestine. For this purpose, floating capsules are formulated, an example of an attractive technological solution [56]. This category includes non-effervescent floating systems; for example, hydrodynamically balanced and non-effervescent tablets, effervescent floating systems, and raft-forming systems [57].

## 8. Hydrodynamically Balanced Systems (HBSs)

This is a flotation form of a drug that floats on the surface of the stomach contents to prolong the release of the drug.

**Figure 6:**
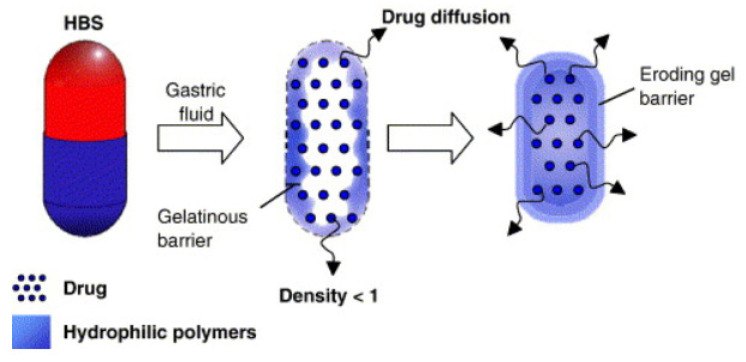
Hydrodynamically balanced system (HBS): Structure of a floating capsule. Mode of action of HBS capsules made of gelatin mass with specific density < 1 [59].

The technology for developing a hydrodynamically balanced system involves mixing drugs with hydrocolloids such as hydroxypropyl methylcellulose (HPMC) K4M, K15M, and K100M. Low-density fatty acid materials and hydrogenated vegetable fats are also used to develop HBSs [57]. These systems are based on the ability to stay in the stomach for hours, resulting in improved dissolution and bioavailability of the poorly soluble drugs in a high pH environment. These systems are also used for the local delivery of drugs to the stomach and proximal small intestine (Figure 6).

Madopar^®^ HBS is a typical example of an HBS capsule containing a combination of levodopa and benserazide that is used to treat Parkinson’s syndrome. Levodopa is a dopamine precursor. In contrast to dopamine, it crosses the blood-brain barrier, where it is transformed into dopamine with the participation of decarboxylase. Levodopa is combined with the peripheral decarboxylase inhibitor benserazide to prevent premature decarboxylation. Levodopa is absorbed almost entirely from the gastrointestinal tract, mainly from the upper part of the small intestine. The biological half-life is 1 h. The produced preparation containing levodopa and benserazide, from which the release of the drug substance is significantly slowed down, ensures its even daily concentration. Extending the gastric residence time is achieved since the slowly soluble drug has a lower density than the chyme. Therefore, this preparation may float on the surface of the digestive tract, and only after complete dissolution of the drug substance does it leave the stomach along with the digestive contents. The HPMC in this formulation is used as the gel-forming polymer and hydrogenated vegetable oil as a low-density fatty excipient [58].

The HBS system for Madopar^®^ is designed so that the benserazide blocking peripheral decarboxylase is released faster, so a more significant dose of levodopa reaches the central nervous system (CNS) [60].

A bilayer floating dosage unit composed of HPMC was formulated by Oth et al., to achieve local delivery of misoprostol at the gastric mucosa level. Using a large capsule increases the gastric retention time as it impedes passage through the pylorus opening [61].

Multiple unit types of floating pills, which generate CO_2_, have also been developed. This system consists of a sustained release pill as a seed surrounded by double layers. The inner layer is an effervescent layer containing sodium bicarbonate besides tartaric acid, and the outer layer is a swellable membrane layer containing PVA, shellac, etc.

Wei et al., developed a bilayer floating gastric retention tablet with cisapride as the active ingredient. Sodium bicarbonate was added to the floating layer. When dipped in simulated gastric fluid, the tablet floated to the surface and the drug was released gradually. The in vitro drug release of this type of bilayer was controlled by an appropriate amount of HPMC in the drug-containing layer. A slower release of drugs depended on a higher HPMC content [62].

Mini-tablets formulated by Rouge et al., achieved buoyancy by swelling the excipient or including a sodium bicarbonate gas-generating agent. The displacement of the atenolol containing mini-tablets was significantly improved by adding a sodium bicarbonate gas-generating agent to the floating layer and wet granulation. Atenolol mini-tablets containing 7% sodium bicarbonate and coated with Eudragit NE30D: RS 70:30 gave adequate buoyancy and a drug release rate over 6 h [63].

## 9. Raft-Forming Systems

The raft-forming system consists of effervescent excipients and gel-forming polymers to achieve flotation and sustained drug release. These systems are tablets or liquids that can gel on contact with gastric fluids due to elevated temperature or a change in pH (e.g., cation-induced gelation). The thick gel produced remains intact for many hours in the stomach, leading to buoyancy and controlled drug release applications. Floating rafts can act as a blockade between the esophagus and the stomach. This property renders these formulations ideal for managing gastric esophageal reflux disease. Raft systems deliver antacids, such as aluminum hydroxide, simethicone, and calcium carbonate [64]. Gaviscon^®^ is a commercial raft-forming formulation that is available as tablets or liquids and is applicable to treat indigestion and heartburn. Its raft-forming mechanism is sodium alginate, sodium bicarbonate, and calcium carbonate. Topalkan^®^ is an antacid formulation containing aluminum and magnesium as active components, while alginic acid is the raft-forming excipient.

## 10. Expandable

Expandable drug delivery systems are designed to increase their size or change their geometry in the stomach to prevent it from leaving the stomach through the pyloric sphincter and, in conclusion, to have a longer gastric retention time [65]. Accordion Pill^®^ is an expandable capsule that contains carbidopa and levodopa as active components to treat Parkinson’s disease. An unfolding mechanism achieves gastric retention through the presence of folded multilayer polymeric films. Hydrophilic polymers, such as HPMC, PEO, and Carbopol^®^, are usually utilized in the swelling expandable systems thanks to their capacity to absorb water and increase their volume by swelling [65,66].

A few different approaches can achieve gastro retentive drug delivery. These include floating, hydrodynamically balanced, raft-forming, or expandable systems, and combinatory approaches of these technologies. Products based on gastro-retentive technologies that are commercially available are summarized in Table 5.

## 11. Long-Acting Oral (LAO) Gastro-Retentive Drug Delivery Systems

Currently used technological solutions are based on 3D printing technology. The superiority of 3D dosing systems over conventional characters is summarized, emphasizing their durable designs. The pioneers of these innovative technologies are the pharmaceutical organizations Lyndra, Assertio, and Intec, which have taken giant steps towards commercializing long-lasting drugs.

Long-acting oral (LAO) gastro-retentive drug delivery systems have the potential to improve patient adherence by improving pharmacokinetic profiles and achieving better clinical responses compared to immediate-release formulations. Prior approaches to gastric residence have been described in a recent review and have included floatation, sedimentation, mucoadhesion, and expansion [67]. LAO dosage forms are required to supply potent and consistent drug release while having physical properties sufficient to withstand the gastro-migratory motor complex (MMC) forces in the harsh chemical environment of the gastrointestinal tract [68]. It also must be compatible with other formulation ingredients in established processes such as hot extrusion (HME). Polycaprolactone (PCL) is a widely used, biocompatible thermoplastic material that has excellent physical and chemical properties for this application. Its low melting point (60–65 °C) allows it to be used in mild conditions, minimizing heat exposure of the mixed drugs in well-established processes such as HME.

**Figure 7:**
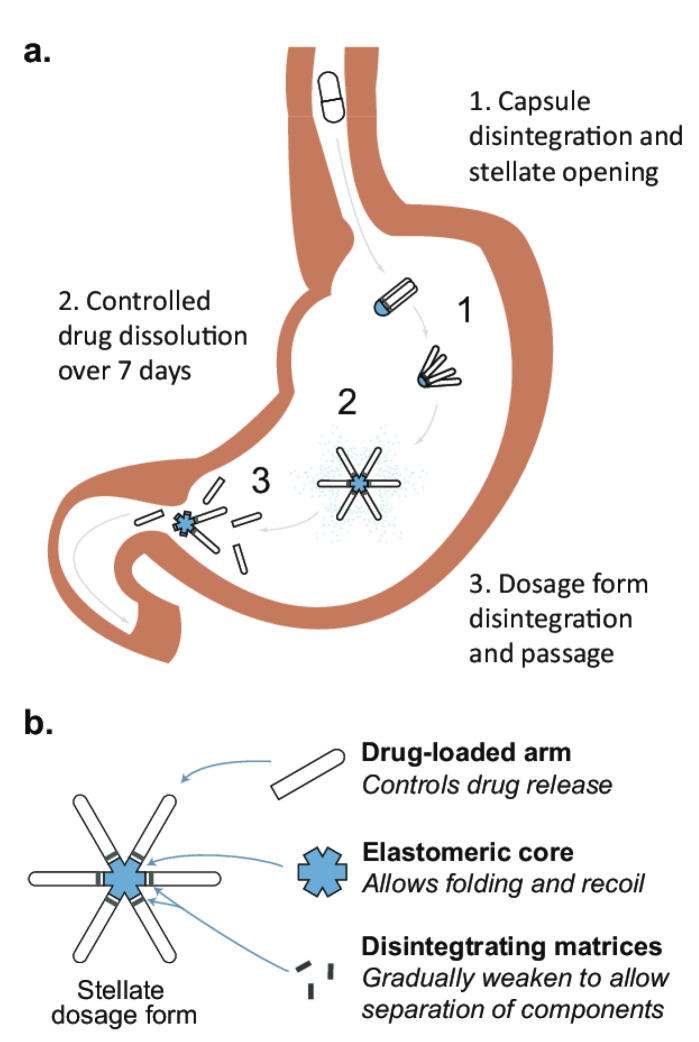
Star-shaped gastric retention dosage form developed by Lyndra (**b**). The stellate dosage form is administered in a capsule. Upon dissolution, the capsule releases a large star that remains in the stomach for approximately 7 days to provide a constant release of the drug (**a**). After the drug is released, the matrix breaks down into smaller components that pass safely through the digestive tract [69].

The new form of the drug, developed by Lyndra, makes it possible to take several substances simultaneously by administering a unique capsule only once a week (Figure 7). The system is made of six polymer arms arranged radially in the shape of a star. It is folded and then placed in a gelatin capsule so that the patient can easily swallow it. The capsule dissolves in the stomach’s acidic environment, and the large star inside it remains in the stomach for about 7 days. At the same time, its creators ensure that it does not block the stomach contents. Each polymer star arm can contain drugs that will be gradually released at the acidic pH of the stomach throughout the week. After a week, the star core that connects all of the arms dissolves, which causes it to break down into parts that leave the stomach without any problems and are digested in the other parts of the gastrointestinal tract [69]. The new therapeutic system has already been tested in animals (pigs) treated simultaneously with antimalarial drugs and three antiviral drugs [70]. In addition, Lyndra has conducted human clinical trials. In one study, eight healthy volunteers were given memantine capsules. Each contained 50 mg of the drug, corresponding to a weekly dose. After 7 days, it was shown that the level of the drug in the body was the same as in patients taking the capsule daily.

However, the Acuform^®^ technology by Assertio is already used in several products sold on the market. Acuform^®^ uses swellable polymers in a tablet that expands when hydrated to achieve gastric retention for 8–10 h. Intec Pharma’s Accordion Pill^®^ is currently in Phase III clinical trials. The pill consists of a multilayer film folded into a capsule that expands as the capsule dissolves to achieve gastric retention for up to 12 h. This allows the dosage form to remain in the stomach, releasing a steady dose of the drug until the material disintegrates and leaves the gastrointestinal tract. Gastro-retentive LAO dosage forms could provide a platform for developing ultra long-acting oral products that benefit patients in many therapeutic areas.

## 12. Hot-Melt Extrusion (HME) and Co-Extruded Solid Solutions

The hot-melt extrusion technique was first invented to manufacture lead pipes at the end of the eighteenth century [50]. This process, widely used since the 1930s in the plastics and food industries, found its application in the pharmaceutical industry in the early 1980s. The idea behind the HME technology is to dissolve or disperse the drug substance in a polymer matrix, thus obtaining many applications. The technology of HME using various polymers to melt it has made it possible to form different shapes that have proven to be a robust method of producing numerous drug delivery systems in the pharmaceutical industry [71]. HME processes are currently used in the pharmaceutical field to produce a variety of dosage forms such as granules, pellets, tablets, suppositories, implants, subcutaneous systems, and ophthalmic inserts. Moreover, technological solutions of HME allow polymer films to show the adhesion phenomenon [72].

The pioneering research by Follonier et al., on hot-melt extrusion technology for the production of pellets based on polymers with the sustained release of various easily soluble drugs contributed to the development of the HME process by Breitenbach in pharmaceutical production [51]. Breitenbach and Lewis developed the Meltrex TM technology in 2003 [73].

Low melting point polymeric carriers or waxes (e.g., hydroxypropyl cellulose, ethylcellulose, methacrylic acid ester copolymers, macrogol copolymers, etc.) are the most commonly used. It is crucial to choose the appropriate carrier due to its influence on the kinetics of the release of substances from the drug form. Suppose water-insoluble polymers are used, i.e., ethylcellulose or waxes (e.g., carnauba wax). In that case, the release of the active substance will take place via diffusion, while the carriers are in the form of soluble macromolecular compounds, i.e., hydroxypropyl cellulose (HPMC), poly (vinylpyrrolidone) or poly (ethylene oxide), will release the drug through matrix diffusion and erosion [74,75].

Advantages of the hot-melt extrusion technique (HME) [71,72,74,75]:

- It does not require the use of water or other solvents (the process is carried out in an anhydrous environment).
- It ensures the continuity of production and product homogeneity.
- A lower number of processing steps and a shorter time to obtain the product compared to traditional technologies.
- Maintains the process efficiency at a high level, compared to classical production methods.
- The possibility of obtaining molecular dispersions and solid dispersions.
- The possibility of producing drug forms containing thermolabile substances.
- The possibility of obtaining amorphous forms.
- Masking the taste and smell of medicinal substances.
- The possibility of shaping a mixture of substances that cannot be compressed and obtaining any shape.

Despite the undoubted advantages of the HME technology, a considerable challenge for the broader use of this method is the risk of degradation of the drug substance and matrix under the influence of the increase in process temperature and the action of shear forces. Overcoming these limitations is possible through the appropriate modification of equipment, process control, and the use of appropriate plasticizers, due to which the process temperature can be reduced (e.g., copolymers of ethylene oxide and propylene oxide, citric acid esters, fatty acid esters, triacetin, short-chain macrogols) [75].

A fascinating technological aspect is the possibility of obtaining an amorphous form of a drug substance in the HME process, which is often characterized by better solubility and bioavailability than the crystalline form. By introducing HME techniques in combination with 3D printing, it is possible to improve the bioavailability and solubility of drugs and obtain long-term prolonged release of the active substance. This possibility is beneficial when medical implants manufactured through 3D printing are considered.

The application of melt extrusion to the manufacturing of tablets is a relatively new technique; despite successes in developing new formulations, the method is complex and technologically demanding. As of now, just four formulations have been successfully registered and manufactured using HME. However, many new formulations are currently under development (Table 6). Kaletra^®^, which contains lopinavir and ritonavir for treating HIV, is the first co-formulated pharmaceutical compound to be successfully tableted using a proprietary melt extrusion process [73]. The successful implementation of Kaletra^®^ demonstrates that hot extrusion overcame the poor solubility and negligible bioavailability of solid oral lopinavir and ritonavir formulations and represents a breakthrough from previous experimental combination drug development efforts.

Co-extrusion is the simultaneous extrusion of at least two materials to obtain a multilayer extrudate. This has advantages, such as coating the inner layer with an outer layer to modify the release or protection of the inner component, and the simultaneous administration of two incompatible drugs as separate layers. Another advantage of this method is developing single-step fixed-dose combination products [74].

The study of polyethylene oxide (PEO) as an extended-release polymeric carrier for a chlorpheniramine maleate (CPM) bar-shaped tablet prepared by HME was first described by McGinity et al. [76]. It was found that PEO erosion and the drug diffusion through the created swollen gel on the tablet surface controlled the release profile of the matrix tablets while the CPM was molecularly dispersed within the PEO matrix [76]. In a study by Dierickx et al., acetylsalicylic acid (ASA) and fenofibrate (FF) were included as hydrophilic and hydrophobic model drugs. Based on screening experiments, the polymers Kollidon^®^ PF 12 and Kollidon^®^ VA 64 were identified as valuable ASA (core) carriers, while Soluplus^®^, Kollidon^®^ VA 64, and Kollidon^®^ 30 were used as FF (coat) carriers [77].

Abdelquader et al. designed a fixed-dose combination drug (FDC) to treat hypertension containing Olmesartan medoxomil and hydrochlorothiazide using co-processing. Both active substances have hydrogen bonding sites and can, therefore, interact during co-processing. This study confirmed such interaction during co-processing and presented a way to inhibit harmful interactions. Co-processing in the presence of both HPMC and an aerosol has been proven to eliminate co-crystallization and minimize drug–drug interaction with subsequent dissolution enhancement [78]. The biggest challenge of the co-extrusion process is finding good combinations of polymers, taking into account pharmaceutical aspects (e.g., good drug release properties), and some technical considerations (e.g., similar extrusion temperature, melt viscosity, interlayer adhesion) [74].

The production of oral drug delivery systems by co-extrusion offers the possibility of combining various drugs with different release profiles, modulating the drug release (either by loading different layers with different amounts of the drug or by introducing the drug into different matrices), and allowing the simultaneous administration of incompatible drugs (composed in separate layers). The only two co-extruded dosage forms available are NuvaRing^®^, a contraceptive vaginal ring, and Implanon^®^, a contraceptive implant [79].

## 13. Three-Dimensional (3D) Printing Technology

Charles Hull is considered a pioneer of 3D printing because, in 1983, he developed, patented, and commercialized the first equipment for printing 3D objects. Hull’s 3D printing technique was based on stereolithography using a laser that moved over the surface of the liquid resin, hardening it. This process was repeated layer by layer many times until the desired shape was formed. In 1988, Charles Deckard filed a patent for selective laser sintering (SLS). The material in the SLS method goes from a solid (powder) through a liquid state and finally back to a solid-state in the form of a sinter [80]. In 1989, Scott Crump filed a patent for fused deposition modeling. In this technique, an object is formed by applying layers of solidifying materials to the desired shape [80,81].

The FDA first approved a 3D printed drug product in August 2015, which opened a new chapter for pharmaceutical manufacturing [80,82]. Over the past 10 years, 3D printing technologies have been developed to address the current limitations in manufacturing medicinal products and the challenges in treating patients. The observed trend of departing from the mass production of drugs towards the design and production of personalized drugs and adjusting the dose to an individual patient’s needs requires optimization of the various 3D printing technologies. Three-dimensional inkjet printing and 3D inkjet powder printing are the two leading technologies used in the pharmaceutical industry. The high degree of flexibility and control, thanks to 3D printing, enables the preparation of dosage forms with many active pharmaceutical ingredients with complex and tailored release profiles. Infinite dosage forms can be created using 3D printing. Tablets with multi-tank printing, microcapsules, nanosuspensions, micropatterns printed with antibiotics, mesoporous, bioactive glass scaffolds, and synthetic extracellular matrices based on hyaluronan, are just some of the innovative dosage forms developed using 3D printing [61]. Three-dimensional printing has recently gained momentum in developing various drug delivery systems, reflected in the exponential increase in published articles and patents [82].

### 13.1. Fused Deposition Modeling (FDM)

The applied material (polymer) is forced through a nozzle heated to its melting point in the FDM method. The nozzle controls the material flow and is moved automatically as instructed by a computer program. The presented technological solution is also desirable when the polymers’ high melting points are used, and the API degradation temperature is similar. Lowering the process temperature translates into maintaining the stability of the API while achieving complete dispersion in the polymer matrix. As with stereolithography, the model is produced layer by layer. In this way, many dosage forms can be made, such as implants, zero-order release tablets, etc., containing the polymer as part of their formulation [83,84]. An FDM-3D printed bilayer oral solid dosage consisting of metformin for prolonged and glimepiride for immediate drug delivery was developed by Gioumouxouzis et al. Metformin and glimepiride were embedded in an Eudragit^®^ RL sustained release layer and a polyvinyl alcohol (PVA) layer, respectively [85]. FDM is the most cost-effective printing technology. A disadvantage of the FDM technique is the limited drug loading capacity and the stability of the thermolabile drugs. Other techniques, such as melt casting, are often combined with FDM to overcome these limitations. Keikhosravi, for the first time, described the preparation of multi-compartment polytablets containing aspirin and simvastatin to permit the physical separation of incompatible drugs by a combination of FDM and melt casting techniques. The simultaneous usage of FDM and melt casting techniques warrant the integrity of polypills by the complete separation of incompatible drugs and their stability [86].

### 13.2. Semi-Solid Extrusion (SSE)

Semi-solid extrusion (SSE) is a subset of 3D printing with material embossing; by sequentially applying layers of paste or gel, it creates objects of any desired size and shape. Compared to other extrusion-based technologies, SSE 3D printing uses low printing temperatures, making it suitable for drug delivery and biomedical applications, and the use of disposable syringes offers advantages in meeting critical quality requirements for pharmaceutical applications [87,88].

### 13.3. Inkjet Printing

Inkjet printing is a powder-based 3D printing method that uses a powder as a substrate onto which different combinations of active ingredients and ink, with different droplet sizes, are sprayed layer by layer, and which eventually solidify into a solid dosage form [83,89,90].

### 13.4. Powder Bed Technology

The potential of 3D printing creates the possibility of combining several active substances in one dosage form, thus limiting the number of drugs taken by the patient and offering the benefit of obtaining an immediate and sustained release of the active substance. It also improves the convenience of use and has a positive effect on adherence to the prescribed dosing schedule, often ensuring comfort for caregivers responsible for administering the drug to children or the elderly. Table 7 presents multi-component formulations and controlled release systems developed using 3D printing technology (3DP).

The ZipDose technology, in addition to the possibility of delivering a large amount of drug with a high degree of dispersion and solubility due to the production of highly porous material, enables the administration of a personalized dose for the patient. The ZipDose technique is based on the multilayer application of a compressed powder saturated with an active substance, which guarantees a quick disintegration of the drug form in contact with a small amount of water. The introduction of ZipDose technology by Aprecia in 2015 and the successful commercialization of Spritam^®^ (levetiracetam), the first 3D printed oral anti-epileptic tablet, took the pharmaceutical industry to the next level in terms of patient treatment options. Children and the elderly often have problems swallowing large-sized tablets several times a day. Yet, failure to take the next dose of the drug may result in a seizure. Produced using a 3D printer and ZipDose technology, Spritam^®^ in the form of an orally disintegrating tablet (ODT), enables the delivery of large amounts of the drug, even up to 1000 mg in a single dose, thus, significantly improving patient comfort [91].

## 14. Conclusions

Progress in applied pharmacy resulted in the spread of more complex drugs, which made it possible to improve the already existing medicinal products due to their innovation. It also initiated the use of compounds that could not be introduced into medicine earlier due to their biological and physicochemical properties. The development of oral dosage forms providing long and sustained release of the API (days or weeks) could transform care, significantly decrease patient burden in chronic disease management, and improve the effectiveness of therapy. Monolithic, multiple-layer, and multiarticulate systems are the most common type of FDCs. Currently, the leading manufacturing techniques utilized in industrial pharmaceutical companies rely on combined wet and dry granulation, hot-melt extrusion coupled with spray coating, and compression of bilayered tablets. Even though 3DP was introduced in the 1980s, there is still much research, especially in creating materials suitable for pharmaceutical and medical applications. Extrusion based on 3D printing is the technology most used to fabricate polypills and customize the dose, dosage form, and release the kinetics, potentially reducing the risk of patient non-compliance [82]. Nowadays, personalized medicines are gaining importance in clinical settings, and 3D printing is highlighted in manufacturing complex and personalized 3D solid dosage forms that could not be manufactured using conventional techniques. One of the latest trends in 3DP is the use of versatile materials that can change their properties under the influence of external factors or over time. Structural modification over time, otherwise known as the fourth dimension, has created a new term known as “4D printing”.

4D printing overcomes the limitations of FDC drugs, such as being unable to adjust the doses of individual components in specific patients, poor bioavailability, or the incompatibility of active substances. An expandable drug delivery system for gastric retention FDCs based on shape memory polymers may be produced by 4DP in a programmable manner through self-folding or self-unfolding, and swelling and deswelling. Moreover, drug release of FDC drugs can be adjusted individually to the altered pH in various gastroenterological diseases in individual patients.

We are convinced that with the elimination of regulatory restrictions related to 4D printing technology, this could be the next technological revolution in fixed-dose combination drugs on the way to personalized medicine.

## Figures and Tables

**Table 1:** Advantages and limitations of fixed-dose combination drugs.

Advantages	Limitations
Complimentary mechanism of action and synergistic effects. Lower doses of individual drugs reduce the frequency of side effects and improve tolerance to treatment.	Less dosage flexibility (it is not possible to change individual doses for each ingredient/Difficulty in dose adjustment.
Fewer tablets and simplification of the dosing schedule (preferably once a day in the morning)	It is best to start and stabilize patients on individual tablets in some situations before starting the corresponding FDC product.
Lower medical costs/Reduced cost. The price of an FDC formulation is usually comparable to, or less than, the total price of the individual components.	Difficulties in identifying the source of possible undesirable actions/Misidentifying the causative medicine when the patient experiences side effects/Some adverse effects are common to many active ingredients, so it may be challenging to identify which medicine is responsible.
Faster achievement of blood pressure control/Glycemic goals (improved glycemic control)/Pain reduction, etc., and treatment targets.	Over- or underdosing. Inadvertent duplication of FDC and single-agent prescriptions occurring.
Convenience/Ease in dispensing. Improves adherence and lowers default rate. Several logistical advantages, such as ordering, planning and drug management.	One of the significant issues with FDCs is the issue of rationality—Several studies from parts of Asia, India, and Latin America have shown that many FDCs are not rational. Furthermore, the government of some countries has banned several FDCs.
Reduction in pill burden/ Simple treatment plan.	The use of one or more broad-spectrum antibiotics in combination (FDC) can also cause serious problems for patients’, e.g., antibiotic-associated diarrhea and increased risk of developing resistance to one or more antibiotics.
All of the above elements improve cooperation between the patient and doctor, which in turn improves effective treatment.	Formulation scientists experience challenges in the development stages of multi-drug formulations, such as compatibility issues among active ingredients and excipients affecting solubility and dissolution.

**Table 2:** Examples of cardiovascular polypills with a marketing authorization and underdevelopment (Adapted from [14,15,16,17]).

Trade Name	Components	Company	Geography	Approval Year
Polypills with marketing approval				
Atamra CV Kit	Atorvastatin, Ramipril, Clopidogrel	Amra Remedies	India	2015
CV-Pill Kit	Ramipril, Metoprolol, Atorvastatin, Aspirin	Torrent Pharmaceuticals	India	2013
Heart Pill	Ramipril, Atorvastatin, Aspirin	Excella Pharma	India	2015
Imprida HCT	Amlodipine, Valsartan Hydrochlorothiazid	Novartis	Austria, Belgium, Bulgaria, Croatia, Czech Republic, Cyprus, Estonia, Finland, France, Germany, Greece, Hungary, Ireland, Italy, Latvia, Lithuania, Luxembourg, Malta, the Netherlands, Poland, Portugal, Romania, Slovakia, Slovenia	2009
Polycap	Aspirin, Ramipril, Hydrohlortiazyd, Atenolol, Simvastatin	Cadila Pharmaceuticals	India and Zambia	2009
Polypill—E	Aspirin, Atorvastatin, Hydrohlortiazyd, Enalapril	Alborz Darou Pharmaceuticals	Iran	2015
Polypill—V	Aspirin, Atorvastatin, Hydrohlortiazyd, Valsartan	Alborz Darou Pharmaceuticals	Iran	2015
Ramitorva	Aspirin, Ramipril, Atorvastatin	Zydus Cadila Healthcare	India	2014/2015
RIL-AA	Ramipril, Atorvastatin, Aspirin	East-West Pharma	India	2014/2015
Starpill	Aspirin, Losartan, Atenolol, Atorvastatin	Cipla	India	2014/2015
Polytorva	Aspirin, Ramipril, Atorvastatin	USV	India	2015
Trinomia Sincronium Iltria	Aspirin, Ramipril, Atorvastatin or Simvastatin	Ferrer	Latin America: Guatemala, Honduras, Dominican Republic, El Salvador, Nicaragua, Argentina, Chile, Paraguay, Ecuador, Mexico; Europe: Belgium, Bulgaria, Germany, Finland, France, Greece, Ireland, Italy, Austria, Poland, Portugal, Romania, Spain, Sweden, Czech Republic.	2014/2015
Triveram	Perindopril, Amlodipine, Atorvastatin	Servier	Austria, Belgium, Bulgaria, Croatia, Czech Republic, Cyprus, Estonia, Finland, France, Germany, Greece, Hungary, Ireland, Italy, Latvia, Lithuania, Luxembourg, Malta, the Netherlands, Poland, Portugal, Romania, Slovakia, Slovenia	2015
Triplixam	Amlodipine, Perindopril Indapamide	Servier	Austria, Belgium, Bulgaria, Croatia, Czech Republic, Cyprus, Estonia, Finland, France, Germany, Greece, Hungary, Ireland, Italy, Latvia, Lithuania, Luxembourg, Malta, the Netherlands, Poland, Portugal, Romania, Slovakia, Slovenia	2014
ZYCAD-4 kit	Ramipril, Metoprolol, Atorvastatin, Aspirin	Zydus Cadila Healthcare	India	2015
Polypills in development				
GSK3074477	Amlodipine, Rosuvastatin	GlaxoSmithKline	-	-
Livalo	Pitavastatin, Valsartan	JW Pharmaceutical	-	-
Unnamed	Valsartan, Rosuvastatin	EMS	-	-

**Table 3:** Various combinations of active pharmaceutical ingredients in bilayer tablets.

Drugs	Dosage Form	Form Rational	Year	Ref. No
Metformin HCl Evogliptin tartrate	Bilayer tablets	Synergistic effect in diabetes	2021	[25]
Metformin HCl Dapagliflozin l-proline	Bilayer tablets	Synergistic effect in diabetes	2021	[27]
Trimetazidine HCl, Clopidogrel bisulphate	Bilayer tablets	Cytoprotective anti-ischemic, platelet inhibitor in acute coronary syndromes	2014	[28]
Metformin HCl, Glimipiride	Bilayer tablets	Synergistic effect in diabetes	2011	[29]
Metformin HCl Atorvastatin Calcium	Bilayer tablets	To develop polytherapy for the treatment of NIDDS and hyperlipidemia	2011	[30]
Piracetam Vinpocetine	Bilayer tablets	Synergistic effect in Alzheimer disease	2011	[31]
Trihexyphenidyl HCl Ziprasidone HCl.	Bilayer floating tablets	For extending the metabolism and improving the bioavailability of both APIs; simultaneous administration of antipsychotics and anticholinergics to prevent or treat extrapyramidal syndrome (EPS)	2011	[26]
Cefixime Trihydrate, Dicloxacillin Sodium	Bilayer tablets	Synergistic effect in bacterial infections	2011	[32]
Amlodipine besylate, Metoprolol Succinate	Bilayer tablets	Synergistic effect in hypertension	2009, 2011	[33,34]
Diclofenac Sodium, Paracetamol	Bilayer tablets	Synergistic effect in pain	2010	[35]
Atenolol, Lovastatin	Bilayer floating tablets	Synergistic effect in hypertension and biphasic release profile	2009	[36]
Montelukast, Levocetrizine	Bilayer tablets	Improvement of the stability of drugs in combination	2009	[37]
Salbutamol Theophylline	Bilayer tablets	Synergistic effect of drugs in asthma	2009	[38]
Rifampicin, Isoniazid	Capsule and tablet in capsule	To avoid interaction b/w incompatible drugs	2007	[39]
Ranitidine, Aspirin	Single-layer coated tablets	Minimizing the contact of two incompatible drugs	2003	[40]
Misoprostol, Diclofenac	Bilayer tablets	To minimize contact b/w drugs	2007	[41]

**Table 4:** FDA- and EMA-approved FDC products with multilayer technology.

Product Name	Chemical Name	Dosage Form	Therapeutic	Manufacturer/ Developer	Ref
Alprax Plus	Sertraline/ Alprazolam	Bilayer tablets	Anti-depressant	Torrent Pharmaceuticals Ltd.	[44]
Glycomet-GP2Forte	Metformin hydrochloride/Glimepiride	Bilayer tablets	Anti-diabetic	USV Limited	[45]
Lopressor HCT	Metoprolol/Hydrochlorothiazide	Bilayer tablets	Antihypertensive	Novartis Pharmaceuticals Corporation	[46]
Diovan HCT	Valsartan/ Hydrochlorothiazide	Bilayer tablets	Antihypertensive	Novartis Pharmaceuticals Corporation	[47]
Lotensin HCT	Benazepril/Hydrochlorothiazide	Bilayer tablets	Antihypertensive	Novartis Pharmaceuticals Corporation	[48]
Clarinex-D	Desloratadine/Pseudoephedrine sulphate	Bilayer tablets	Allergic rhinitis	MSD	[49]
Treximet	Sumatriptan/Naproxen sodium	Bilayer tablets	Migraine	Pernix Therapeutics	[50]
Atripla	Efavirenz/Emtricitabine/Tenofovir disoproxil fumarate	Multilayer tablets	HIV/AIDS	Gilead Sciences	[51]
Under development -In clinical trials/Patent EP 2 682 112 A1	Flurbiprofen 100 mg Famotidine 20 mg	Multilayer tablet	An NSAID with anti-inflammatory, analgesic and antipyretic activities, an H2 receptor antagonist used for preventing or minimizing gastroin-testinal side effects caused by NSAIDs	Pharmaceutical Research Unit, Jordan	[52]

**Table 5:** Available commercial expandable products.

Delivery System	Brand Name	Active Pharmaceutical Ingredient	Manufacturing Company
Hydrodynamically Balanced	Madopar^®^	Levodopa and Benserazide	Intec Pharma (Israel)
Raft-forming	Gaviscon^®^ Tablets	Sodium bicarbonate and Calcium carbonate	Reckitt Benckiser Healthcare (UK) Ltd.
	Topalkan^®^	Aluminum and magnesium	Pierre Fabre Medicament (France)
Expandable	Accordion Pill^®^	Levodopa and Carbidopa	Intec Pharma (Israel)
	Janumet^®^ XR	Sitagliptin and Metformin	Merck Sharp & Dohme (USA)

**Table 6:** Available commercial products processed via HME.

Product	Company	HME Purpose	Indication	Approval Year
Kaletra (Ritonavir, Lopinavir)	AbbVie	Amorphous dispersion	Anti-viral (HIV)	2000
Viekira Pak (Ombitasvir, Paritaprevir, Ritonavir, dasabuvir)	AbbVie	Amorphous dispersion	Anti-viral (HCV)	2014
Zok-Zid tablet (Metoprolol tartrate, hydrochlorothiazide)	Pfizer	Hot-melt co-extrusion	Hypertension	2012
NuvaRing (Etonogestrel, Ethinyl Estradiol)	Merck	Shaped system	Contraceptive	2001
Eucreas (Vildagliptin, Metformin HCl)	Novartis	Melt granulation	Diabetes	2008
Dapivirine, Maraviroc, BMS793, CMPD 167	Particle sciences	Shaped system	Anti-viral (HIV)	Under development

**Table 7:** Pharmaceutical preparations in the development stage—obtained by 3DP technology.

3D Printing Technology Used	Formulations	API(s)	Excipient(s)	Year	Ref. No
Extrusion	Immediate/sustained polypill	Aspirin, hydrochlorothiazide, ramipril, pravastatin sodium, atenolol	Cellulose acetate, D-mannitol, polyethylene glycol (PEG 6000) sodium starch glycolate, polyvinylpyrrolidone (Povidon K30), hydroxypropyl methylcellulose (Methocel™ K100MCR), lactose	2015	[88]
Semi-solid extrusion (SSE)	Multiactive tablets (polypill)	Nifedipine, Glipizide, Captopril	Hydroxypropyl methylcellulose (HPMC 2208), polyethylene glycol (PEG 6000), tromethamine, lactose, sodium chloride, D-mannitol, croscarmellose sodium, microcrystalline cellulose, sodium starch glycolate, hydroxypropyl methylcellulose (Methocel™), cellulose acetate	2015	[92]
Stereolithography (SLA)	Modified tablets	4-Aminosalicylic acid and paracetamol	Polyethylene glycol diacrylate, diphenyl (2,4,6-trimethylbenzoyl) phosphine oxide, and (PEG 300)	2016	[93]
Fused deposition modeling (FDM)	Caplets	Paracetamol and caffeine	Polyvinyl alcohol	2016	[94]
	Mini-tablets (for pediatric use)	Caffeine and propranolol	Hyprolose and Hypromellose	2021	[95]
	Bilayer dosage form	Metformin and Glimepiride	Eudragit^®^ RL, polyvinyl alcohol (PVA)	2018	[85]
	Shell-core tablets	Theophylline, budesonide, and diclofenac sodium	Core: Polyvinylpyrrolidone, triethyl citrate, talc or tribasic phosphate sodium, and APIShell: Eudragit^®^ L 100–55, triethyl citrate, and talc	2017	[96]
	Bilayer tablet	Enalapril maleate, and hydrochlorothiazide	Methacrylate polymer	2018	[97]
Inkjet 3D printing	Multidrug implant	Rifampicin and isoniazid	Polyethylene oxide, polylactic acid (PLA), polyvinyl alcohol (PVA)	2009	[98]

## Data Availability

Not applicable.
